# Lipoprotein-Induced Increases in Cholesterol and 7-Ketocholesterol Result in Opposite Molecular-Scale Biophysical Effects on Membrane Structure

**DOI:** 10.3389/fcvm.2021.715932

**Published:** 2021-07-16

**Authors:** Manuela A.A. Ayee, Irena Levitan

**Affiliations:** ^1^Department of Engineering, Dordt University, Sioux Center, IA, United States; ^2^Division of Pulmonary and Critical Care, Department of Medicine, University of Illinois at Chicago, Chicago, IL, United States

**Keywords:** low-density lipoprotein, oxysterol, multicomponent phospholipid bilayer, molecular dynamics simulation, lipid order, sterol tilt, hypercholesterolemia, mass spectrometry

## Abstract

Under hypercholesterolemic conditions, exposure of cells to lipoproteins results in a subtle membrane increase in the levels of cholesterol and 7-ketocholesterol, as compared to normal conditions. The effect of these physiologically relevant concentration increases on multicomponent bilayer membranes was investigated using coarse-grained molecular dynamics simulations. Significant changes in the structural and dynamic properties of the bilayer membranes resulted from these subtle increases in sterol levels, with both sterol species inducing decreases in the lateral area and inhibiting lateral diffusion to varying extents. Cholesterol and 7-ketocholesterol, however, exhibited opposite effects on lipid packing and orientation. The results from this study indicate that the subtle increases in membrane sterol levels induced by exposure to lipoproteins result in molecular-scale biophysical perturbation of membrane structure.

## Introduction

Elevated blood level of low-density lipoproteins (LDL) is a major risk factor for the development of cardiovascular disease, but the impact of LDL on membrane cholesterol and consequently on membrane structure remain poorly understood. Earlier studies found that exposure to high levels of LDL does not result in significant cholesterol loading of macrophages (1, 2), leading to the hypothesis that cholesterol loading that is observed under hypercholesterolemic conditions *in vivo* should be a result, not of LDL uptake, but of its oxidized modifications (oxLDL) (3). Our studies, however, contradicted this hypothesis by showing that no cholesterol loading is observed in endothelial cells exposed to oxLDL (4, 5). We found instead that exposure to oxLDL results in an incorporation of oxysterols into endothelial cells, particularly 7-ketocholesterol, which was also confirmed in aortas of hypercholesterolemic mice (6). The amount of 7-ketocholesterol, however, constituted only a small fraction (<10%) of total tissue sterol, even in highly hypercholesterolemic mice (6). Most recently, we showed that exposing endothelial cells to elevated levels of LDL, which are observed in highly hypercholesterolemic patients, does result in a significant increase in cellular cholesterol. However, the change is a mild increase of ~10 mol% (7). The question therefore arises whether these subtle changes in cellular sterol compositions can have a significant impact on the biophysical properties of the membrane bilayer and membrane structure.

In this study, we address this question by molecular modeling based on mass spectrometry analyses of the cholesterol and 7-ketocholesterol composition of endothelial cells exposed to normal or elevated levels of LDL or oxLDL. Coarse-grained molecular dynamics simulations are performed to investigate the impact of subtle increases in cholesterol and 7-ketocholesterol concentrations on the structural and dynamical properties of multicomponent lipid bilayer membranes.

## Materials and Methods

### Experimental Procedures

#### LDL Isolation and Oxidation, Cell Culture, and LDL/oxLDL Exposure

LDL was isolated from human plasma obtained from a local blood bank—Lifesource (now Vitalant, Chicago, IL), as described in our previous studies (7). Briefly, LDL was isolated by sequential centrifugation in potassium bromide (KBr, Acros Organics; Thermo Fisher Scientific, Waltham, MA), and dialyzed to remove KBr and EDTA. Oxidized LDL (oxLDL) was prepared by Cu^2+^ as previously described (8). The degree of oxidation was assessed by the TBARS (thiobarbituric acid reactive substances) content of LDL and was measured using a TBARS Assay Kit (ZeptoMetrix, Buffalo, NY) as expressed with malondialdehyde (MDA) equivalent. Experiments were performed with Human Aortic Endothelial cells (HAECs; Lonza, Allendale, NJ), grown in standard conditions. Serum-starved cells were treated with either 10 μg/ml oxLDL, 50 mg/dl LDL, or 250 mg/dl LDL for 24 h, then lysed in methanol and stored at −80°C.

#### Mass Spectrometric Analysis of Lipids

Cell lipids were extracted by a modified Bligh and Dyer procedure with the use of 0.1 N HCl for phase separation, as described by Berdyshev et al. (9). During the initial step of lipid extraction, internal deuterated standards were used (cholesterol-d7, 7-keto-cholesterol-d7) (Avanti Polar Lipids, Alabaster, MO). Extracted lipids were dissolved in methanol/chloroform (4:1, v/v) and aliquots taken to determine the total phospholipid content. Sequential liquid chromatography-electrospray ionization-tandem mass spectrometry (LC-ESI-MS/MS) analyses were performed in the Mass Spectrometry Core at National Jewish Health, as previously described (8).

#### Bio-orthogonal Cholesterol Sensor

A novel bio-orthogonal cholesterol sensor was used to simultaneously measure cholesterol levels separately in the outer and inner membrane leaflets (10). Briefly, a cholesterol-binding protein was tagged with a fluorescent dye that changed color when the protein bound cholesterol. The color-change indicated the ratio of bound to free cholesterol, giving an indication of the spatial distribution of cholesterol in the membrane leaflet.

### Molecular Dynamics Simulations

**Figure 1:**
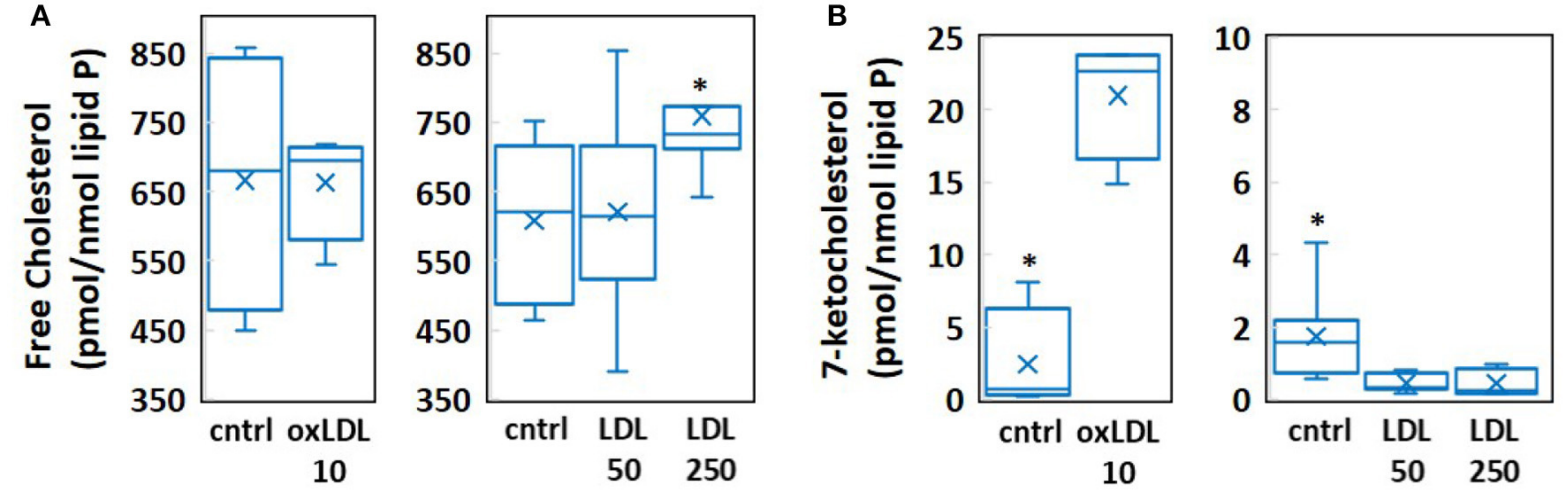
Results of mass spectrometric measurement of **(A)** free cholesterol and **(B)** 7-ketocholesterol content in human aortic endothelial cells (HAECs) treated with 10 μg/ml oxLDL, 50 mg/dl LDL, or 250 mg/dl LDL. (*n* = 4-8, ^*^P < 0.05).

We employed coarse-grained molecular dynamics (MD) simulations to determine the impact of physiologically relevant concentration increases in cholesterol and 7-ketocholesterol on the structural and dynamical properties of multicomponent lipid bilayer membranes. To this end, we created four model systems with sterol concentrations determined by mass spectrometric analyses (Figure 1). These analyses show that exposure to 10 μg/ml oxLDL, which constitutes a high concentration of oxLDL, as compared to the levels detected *in vivo* (11), has no effect on endothelial cholesterol (Figure 1A—left) but results in a significant, almost 10-fold increase in 7-ketocholesterol concentration (from 2.41 to 21.03 pmol/nmol lipid P) seen in Figure 1B—left. In contrast, exposure to clinically relevant levels of LDL, which correspond to pronounced hypercholesterolemia in humans, results in a relatively small (~20%) but significant increase in endothelial cholesterol (Figure 1A—right), and no increase at all in 7-ketocholesterol concentration (Figure 1B—right). Based on the sterol and oxysterol content given in pmol/nmol of lipid phosphate obtained by the mass spectrometric analyses of HAECs, we calculated the cholesterol and 7-ketocholestrol mol% concentrations in each system in the following way: The values of cholesterol content obtained by mass spectrometry for cells exposed to 0, 50, or 250 mg/dl LDL were 608, 621, and 760 pmol/nmol lipid P, respectively (Figure 1A—right). These values correspond to 38 mol% cholesterol for LDL 50 [calculated as 621 chol/(1621 total lipid + chol)] and 43 mol% for LDL 250, assuming that the membrane consists of cholesterol and phospholipids only. Thus, an approximate 22% increase in the absolute cholesterol content from LDL 50 to LDL 250 translates into ~13% increase in the mol% ratio between these two systems. It is important to note here that the cholesterol mol% values obtained for each system are likely to be overestimations because they do not take into account any other membrane lipids other than phospholipids and sterols.

From these mass spectrometric results, we created a control bilayer system composed of the lipid species phosphatidylcholine (PC) and sphingomyelin (SM), with 40% cholesterol (CHOL) and 0.24% 7-ketocholesterol (7KC) distributed evenly between both leaflets. We further created three bilayer systems corresponding to HAECs exposed to 10 μg/ml oxLDL, 50 mg/dl LDL, and 250 mg/dl LDL, and containing 2.1% 7KC (with 40% CHOL), 38 and 43% CHOL (with 0.04% 7KC), respectively (Table 1).

**Table 1:** Membrane lipid compositions in four multicomponent bilayer simulation systems with cholesterol and 7-ketocholesterol concentrations obtained from mass spectrometric measurements.

**Membrane Lipid**	**Control**	**oxLDL 10 μg/ml**	**LDL 50 mg/dl**	**LDL 250 mg/dl**
POPC (PC)	33%	32%	35%	32%
DPPC (PC)	8%	8%	9%	8%
Sphingomyelin (SM)	18%	17%	19%	17%
Cholesterol (CHOL)	40%	40%	38%	43%
7-ketocholesterol (7KC)	0.24%	2.06%	0.04%	0.04%
Total CG lipids + sterols	8,446	8,448	8,446	8,444
Total CG water	110,697	111,023	110,405	111,327

For the simulations, coarse-grained (CG) molecular topologies of all lipid and sterol species were obtained from or developed based on the MARTINI 2 force field version 2.0 (12, 13). CG topologies of POPC (v2.0_POPC_02), DPPC (v2.0_DPPC_01), SM (v2.0_DPSM_01), cholesterol (v2.0_CHOL_02), and water (v2.0) were obtained from the MARTINI website (http://cgmartini.nl/). We developed a topology for 7-ketocholestol by adapting the standard Martini 2.0 cholesterol virtual sites topology developed by Melo et al. (14) and replacing the third SC3 interaction site with a heavier, and more polar SNa bead to represent the ketone moiety as shown in Table 2. This 7KC topology was validated by calculating an octanol-water partition free energy (ΔG = 71.1 ± 1.0 kJ/mol) using the Bennett Acceptance Ratio (BAR) method (15) as previously described (14). As expected for a more polar molecule, our calculated partition free energy value for 7KC is lower than the ΔG of 86.7 ± 0.9 kJ/mol calculated by Melo et al. for the virtual site cholesterol topology. Our 7KC topology was further validated by comparison of the sterol tilt angle distribution to distributions from atomistic simulations (see *Sterol Orientation* section). Interaction sites in the MARTINI force field are formed by grouping two-four heavy atoms into one bead, with the underlying physicochemical characteristics of the molecules preserved by the properties assigned to each interaction site. Although CG topologies of membrane components, as opposed to atomistic structures, result in a small loss of detail, the MARTINI force field is parameterized against both atomic-level simulations and experimental data. The structural changes we investigated in this work occurred on length- and timescales that have been previously well-probed by CG simulations (16–18).

**Table 2:** Lipid topology developed for 7-ketocholesterol based on Martini 2.0 cholesterol model with virtual sites (14).

**Particle number**	**Martini type**	**Site name**	**Mass**
1	SP1	KOH	77.22
2	SC1	K1	0
3	SNa^*^	K2	52.66
4	SC1	K3	159.65
5	SC1	K4	0
6	SC1	K5	0
7	SC1	KC1	39.44
8	C1	KC2	72

* *The modified interaction site*.

**Figure 2:**
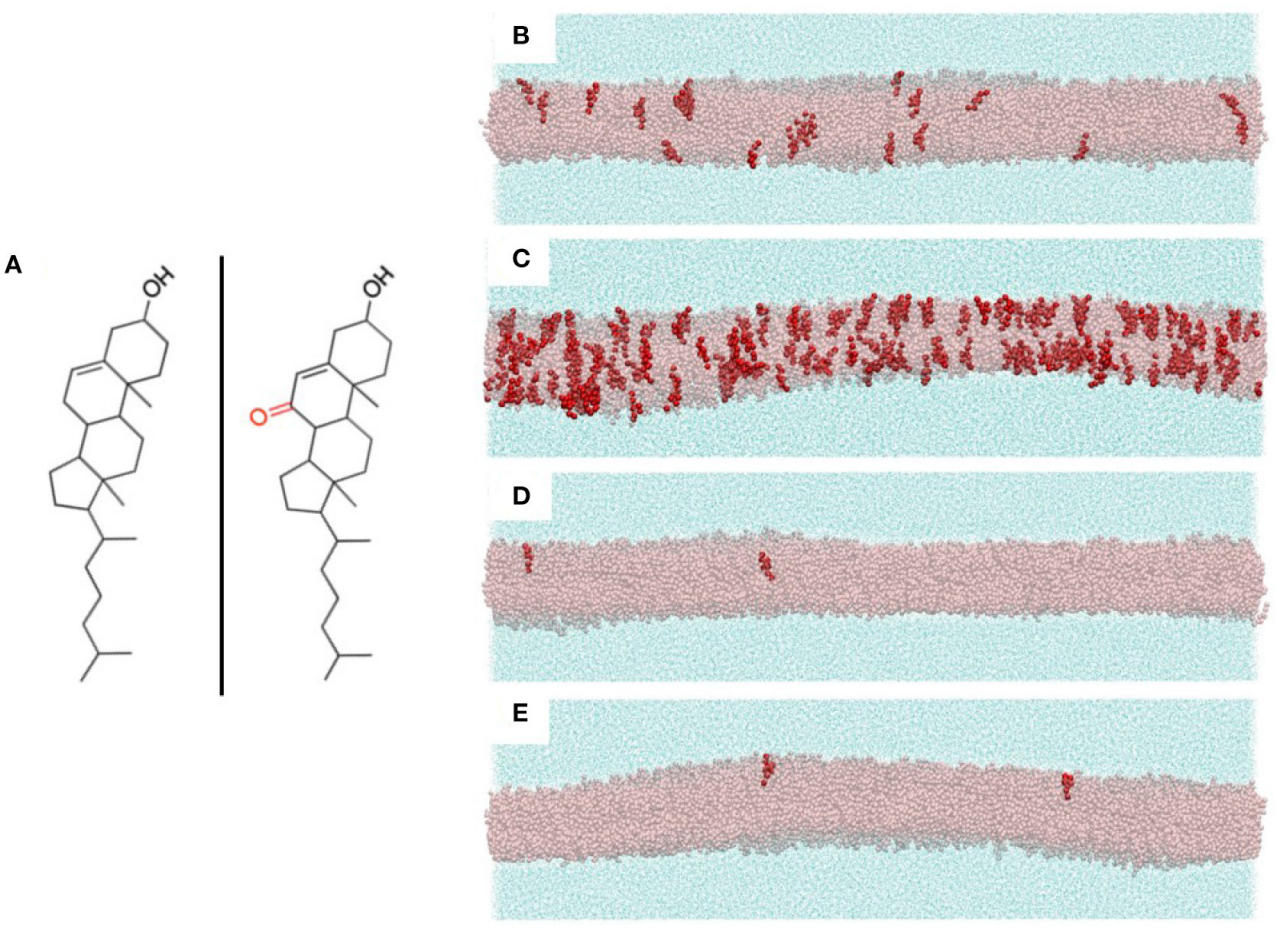
**(A)** Chemical structures of cholesterol (left) and 7-ketocholesterol (right) showing the locations of the hydroxyl groups and the ketone moiety on the oxysterol. **(B-E)** Snapshots of the four multicomponent simulation systems with all bilayer lipids hidden except cholesterol (transparent pink representation) and 7-ketocholesterol (red opaque representation). Surrounding each bilayer are water particles (cyan solvent representation). **(B)** control system (40% CHOL, 0.24% 7KC); **(C)** oxLDL 10 μg/ml (40% CHOL, 2.1% 7KC); **(D)** LDL 50 mg/dl (38% CHOL, 0.04% 7KC); **(E)** LDL 250 mg/dl (43% CHOL, 0.04% 7KC).

The compositions of the PC and sphingomyelin components of our multicomponent bilayer models were chosen to reflect the major constituent lipids of endothelial membranes. Specifically, PC and SM concentrations in human umbilical vein endothelial cells (HUVECs) have been found to be 54 and 23%, respectively (19). Although a large assortment of phospholipids is known to be present in endothelial cell membranes, the acyl tails of these lipids primarily contain saturated palmitic and stearic fatty acids and unsaturated oleic and arachidonic fatty acids (20, 21). Based on this, we created four multicomponent bilayers with the PC/SM component composed of 70% of the combined PCs dipalmitoylphosphatidylcholine (DPPC), with two saturated palmitic tails, and 1-palmitoyl-2-oleoylphosphatidylcholine (POPC), with one palmitic tail and an unsaturated oleic tail. Of these PCs, we chose 80% to be POPC and 20% DPPC. The remaining 30% of our PC/SM component was composed of the SM: N-stearoyl-D-erythro-sphingosylphosphorylcholine, with one saturated stearic tail and an unsaturated sphingosine chain. The control bilayer contained 33% POPC, 8% DPPC, and 18% SM molecules, resulting in a total of almost 8,500 total phospholipids and sterols evenly distributed between both leaflets. Each simulation system consisted of a central, multicomponent bilayer, surrounded on both sides by CG water particles (~111,000 total water particles, with each CG water particle corresponding to four real water molecules). The chemical structures of cholesterol and 7KC are shown in Figure 2A, left and right, respectively. Snapshots of equilibrated simulation boxes for each multicomponent system are shown in Figures 2B–E.

MD simulations were performed using the GROMACS 2019. Two molecular dynamics simulation package (22–26) and the straight-cutoff Lennard-Jones potential scheme with potential modifiers described by de Jong et al. (27). Graphics were rendered using the Visual Molecular Dynamics program (28). All simulations were periodic in all directions and run with a timestep of 30 fs for at least 2.5 μs at physiological temperature (310.15 K), with the first microsecond used as equilibration. A shifted (0.9 nm to a cutoff at 1.1 nm) Lennard-Jones 12-6 potential was used to describe van der Waals interactions between pairs of neutral, non-bonded particles. A Coulombic electrostatic potential was used for charged, non-bonded interactions and shifted from 0 nm to a cutoff at 1.1 nm. Long-range electrostatic interactions were neglected. A weak harmonic potential was used between covalently bonded particles, and a weak harmonic cosine potential was used for angles between three consecutive bonded particles. A time step of 30 fs was used with a velocity Verlet algorithm (29) to integrate the equations of motion (30–32). An isothermal-isobaric (NPT) ensemble was employed in each simulation system. Each system component was coupled separately to a temperature bath with a coupling constant of 1.5 ps using a velocity rescale thermostat (33). Pressure was maintained in a semi-isotropic state at 1.01 bar using a Parrinello-Rahman barostat (30) with a 12 ps coupling constant, to maintain a tensionless bilayer. The velocity of the center of mass was removed every 100 timesteps before temperature calculation to prevent the systems from freezing and translating through space as an artifact of round-off error accumulation by the numerical integration method (34).

[Statistical Analyses: *P*-values were calculated from Student's *t*-tests, with *P-*values < 0.05 considered significant.]

## Results

Although the increases in cholesterol and 7-ketocholesterol concentrations, as compared to the control system, were relatively subtle, we observed distinct and significant changes in the structure and dynamics of each bilayer membrane over the course of our simulations. We will present, herein, changes in membrane biophysical properties that were observed in response to these small, but physiologically relevant increases in sterol and oxysterol content.

### Changes in Bilayer Lateral Area and Thickness

**Figure 3:**
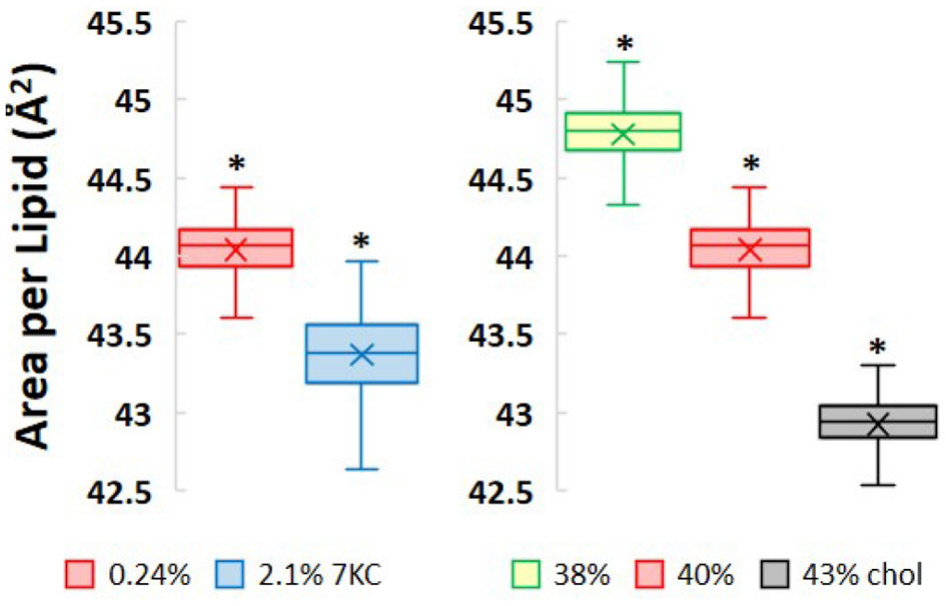
Area per lipid values measured as lateral area of each simulation system divided by total number of lipids over 1.5 μs of simulation time. (*P*-values calculated using unpaired Student's *t*-test. *P < 0.05).

Incorporation of increasing levels of both cholesterol and 7-ketocholesterol at constant surface pressure causes the bilayers to condense laterally as shown in Figure 3. A 10-fold increase in 7KC content from 0.24 to 2.1% causes a very small, but significant decrease in bilayer lateral area of 1.6% (i.e., from 44.0 ± 0.01 Å^2^/lipid to 43.4 ± 0.01 Å^2^/lipid). This small change in lateral area is not accompanied by a corresponding change in bilayer thickness, measured as the distance between the phospholipid phosphate headgroups in each leaflet (~43 Å in both 7KC systems). Subtly increasing levels of cholesterol, on the other hand, have a larger lateral condensing effect on the bilayers, as expected. Increasing cholesterol content from 38 to 40% causes a decrease in lateral area of 1.7%, whereas an additional increase in cholesterol content to 43% causes a 4.2% decrease in lateral area. These decreases in lateral area as cholesterol content increases are accompanied by overall thickening of the bilayer in the direction normal to the water/lipid interface, from 41 Å at 38% cholesterol to 44 Å at 43% cholesterol.

### Decreases in Apparent Lateral Diffusion Coefficients

Increasing sterol concentrations, resulting in lateral condensation of the bilayers, further impacts the dynamic motion of each individual species within the membranes, as measured by lateral diffusion coefficients. The self-diffusion coefficient *D* of a particular species is obtained by the long-time behavior of its mean square displacement (MSD). The lateral diffusion coefficient of each species can, therefore, be defined using continuum diffusion theory as (35).

(1) $$\begin{array}{l} D=\frac{1}{4}\underset{t\to \infty }{\lim}\frac{d}{dt}\left[MSD\left(t\right)\right] \end{array}$$

**Figure 4:**
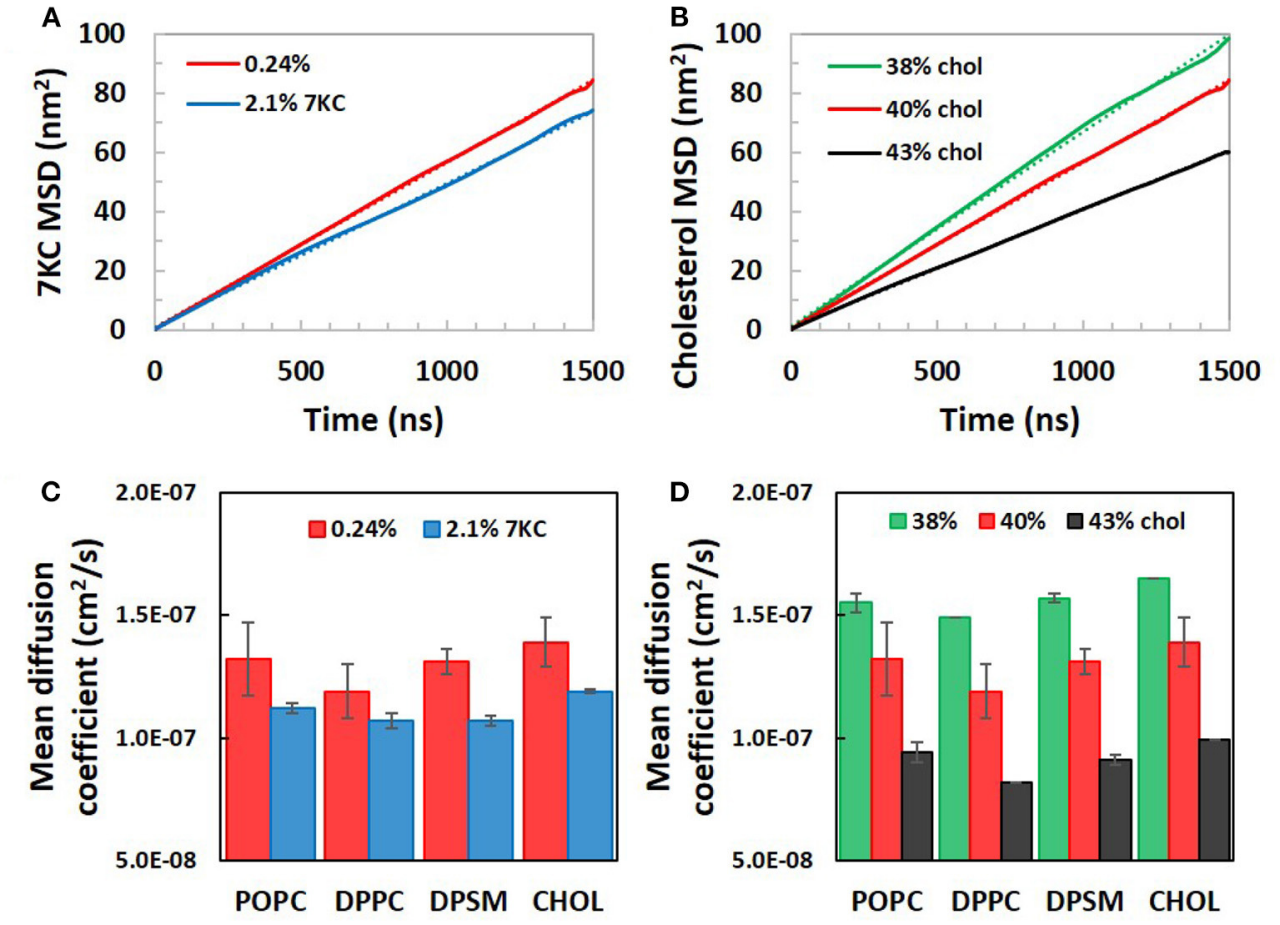
Calculated lateral mean squared displacements of **(A)** 7-ketocholesterol and **(B)** cholesterol in simulation system with increasing 7KC and cholesterol content, along with linear fits to each plot (dotted lines). Linear fits were used to calculate average apparent lateral diffusion coefficients for all species in systems with increasing concentrations of 7KC **(C)** and cholesterol **(D)**. (Diffusion coefficient data shown as time- and ensemble-averaged mean ± standard deviation).

Figures 4A,B present plots of sterol MSD vs. simulation time for bilayer systems with increasing 7KC content (A) and cholesterol content (B). From the slopes of the long-time behavior of similar MSD plots for each lipid species (and cholesterol), average apparent lateral diffusion coefficients were calculated (Figures 4C,D). We note that the absolute values of diffusion coefficients are known to be overestimated in coarse grained simulations due to CG dynamics being faster than atomistic dynamics (36). Therefore, our calculated apparent lateral diffusivity values are slightly larger than expected experimental values for each lipid species (37). For each species, an increase in 7KC or cholesterol content from baseline results in a corresponding decrease in lateral diffusivity, mirroring the trends we observed in lateral area condensation. The lateral diffusion of species within the bilayer may be directly influenced by changes in lipid packing and membrane fluidity.

### Sterol Orientation

**Figure 5:**
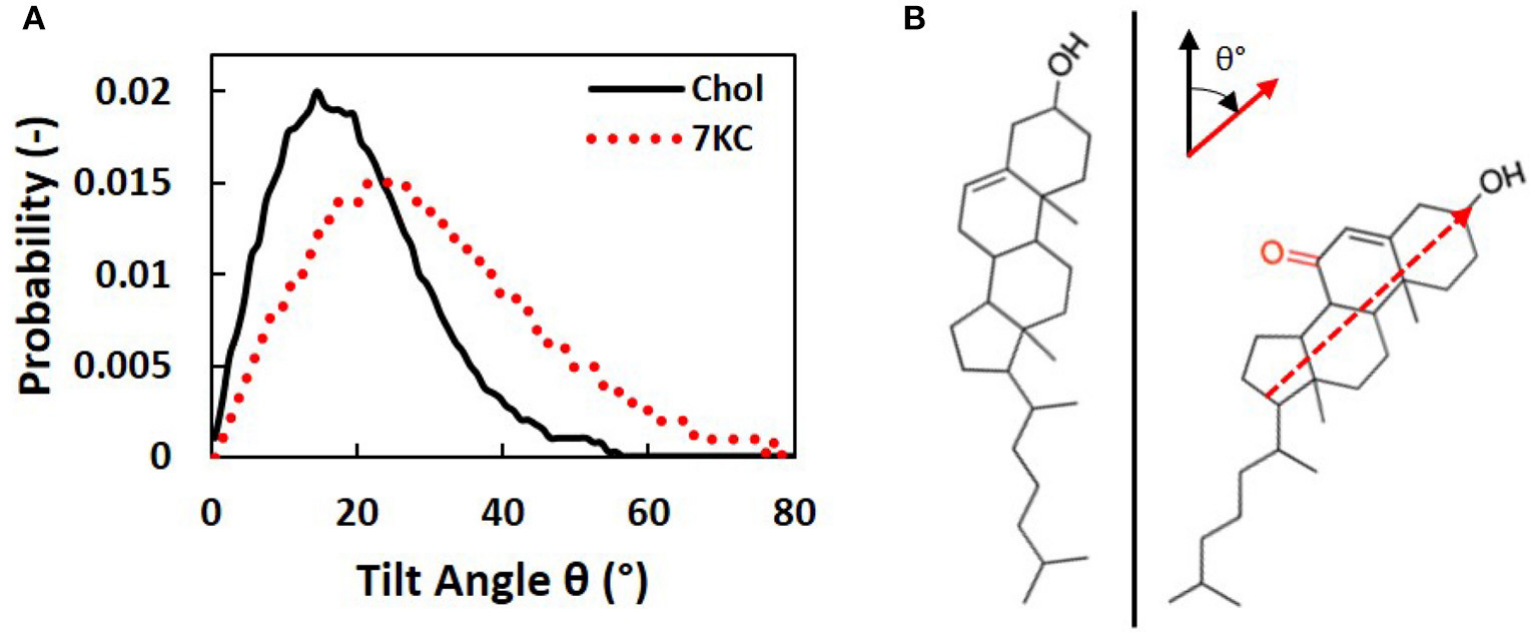
**(A)** Sterol tilt angle probability distributions for cholesterol (solid black curve) and 7KC (dotted red curve), measured with respect to the normal to the bilayer membrane. **(B)** Schematic illustrating an example of the orientational tilt of 7KC (right) compared to cholesterol (left). The vector used to calculate sterol ring tilt angles (θ) is overlaid on the 7KC structure and axes describing the orientation of this vector (red) relative to the bilayer normal (black) are shown.

We calculated tilt angles for the sterol ring structure with respect to the normal to the bilayer membrane, in order to determine the differences in orientation between cholesterol and 7KC molecules in each simulation system. The tilt angle is defined between the normal to the bilayer and a vector from 7KC interaction site K5 to KOH. Similarly, for cholesterol, the vector is defined from site R5 to ROH [see Melo et al. (14) for cholesterol site definitions]. This vector approximates the carbon-17 to carbon-3 vector defined by Kulig et al. (38), which is used to determine sterol ring orientation in the lipid bilayer. Figure 5A shows the tilt angle probability distribution for cholesterol molecules in all systems (solid black curve) compared to the tilt angle probability distribution for 7KC molecules in the 2.1% 7KC system (dotted red curve). These distributions depict the ensemble- and time-averaged range of orientations observed for each sterol over the course of the simulations. The oxidation in the sterol ring of 7KC results in reorientation of the molecules, resulting in overall larger tilt values. The tilt angle probability distribution for 7KC is wider and shifted toward larger angles, indicating that 7KC molecules generally prefer to assume a more tilted orientation within the bilayer membrane as compared to cholesterol (Figure 5B). This phenomenon has been previously predicted for 7KC and other oxysterols in smaller (128 molecule) atomistic MD simulations of single-component POPC bilayers containing 10% sterol concentrations (38). Our tilt angle distributions for both cholesterol and 7KC are similar to those from the atomistic simulations performed by Kulig et al. (38), and therefore serve as a validation check of the behavior of our coarse-grained topology for 7KC within the lipid bilayer.

### Impact on Lipid Order

The structural and dynamical changes we observed, including membrane lateral condensation, bilayer thickening, as well as reorientation of 7KC, all impact the dynamics of the acyl tails forming the hydrophobic core of the lipid bilayers, thereby changing the membrane packing and conformation of the tails. The orientational order parameter (*P*) of the CG pseudobonds in the lipid tails quantifies their alignment and orientation with respect to the bilayer normal (39):

(2) $$P=-\langle \frac{3\cos^{2}\theta -1}{2}\rangle$$

where θ is the angle between each inter-particle bond and the bilayer normal. The angled brackets represent a time- and ensemble-average. Decreasing values of *P* indicate decreased lipid tail packing (greater disorder).

**Figure 6:**
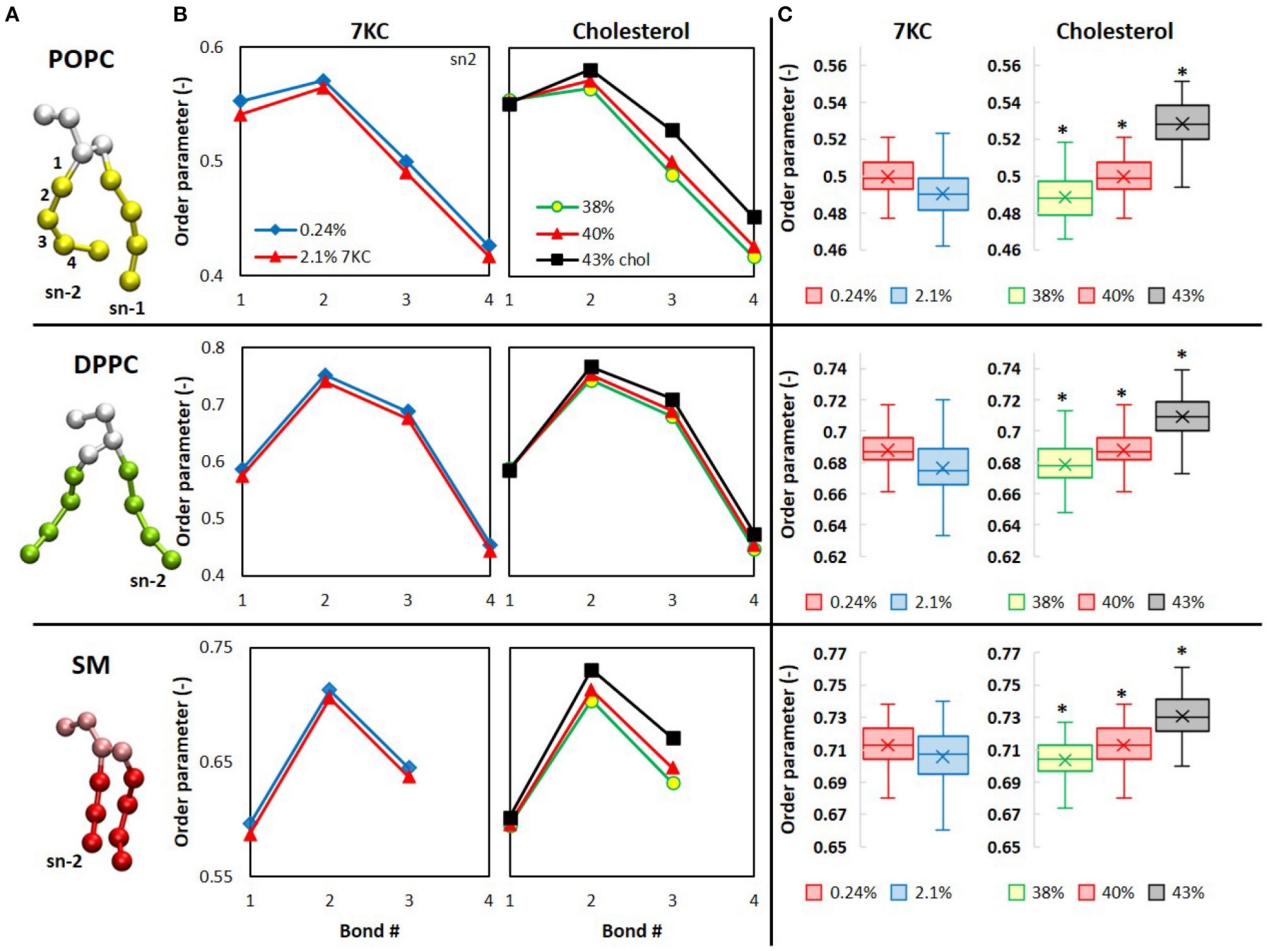
Computational analysis of lipid order in cholesterol and 7KC-contianing bilayers. **(A)** CG structures of each phospholipid are depicted with tails colored differently than headgroups and glycerol backbones. Bond numbers from **(B)** are indexed for the POPC structure (top). **(B)** Orientational order parameters (*P* in Eq. 2) of phospholipid sn-2 tail pseudobonds for simulation systems containing 0.24% 7KC (blue diamonds), 2.1% 7KC and 40% cholesterol (red triangles), 38% cholesterol (yellow circles), and 43% cholesterol (black squares). **(C)** Order parameters for the third pseudobond of each phospholipid sn-2 tail. (*n* = ~6,000 lipid molecules for each bilayer; *P < 0.05).

In Figure 6A, the CG representation of each membrane lipid is illustrated, with the tail bonds labeled to correspond with the order parameters in Figure 6B, which shows the time- and ensemble-averaged order parameters for each pseudobond in the sn-2 tails of each lipid. From Figure 6B, we observe that, for the system containing 2.1% 7KC, the presence of a higher concentration of tilted 7KC molecules causes the order parameters of all bonds in the lipid tails of each of the three bilayer membrane lipid components to decrease (i.e., POPC, DPPC, and SM), as compared to the control system containing 0.24% 7KC. Although this observed decrease in order parameters is not statistically significant, the trend is conserved for all bonds, as indicated by the box plots in Figure 6C, showing the reduction in order of the third pseudobond of the sn-2 tail of each lipid. Conversely, for increasing concentrations of cholesterol, statistically significant increases in order parameter are observed for all sn-2 tail bonds in each lipid species (Figures 6B,C). [We note here that similar trends are observed with the sn-1 tails of all lipids (data not shown)]. As expected, the order parameters for each lipid in the 43% cholesterol system are highest, indicating that this system experiences the greatest lipid tail alignment and the least fluidity.

### Cholesterol Content Measured by Novel Bio-Orthogonal Sensors

**Figure 7:**
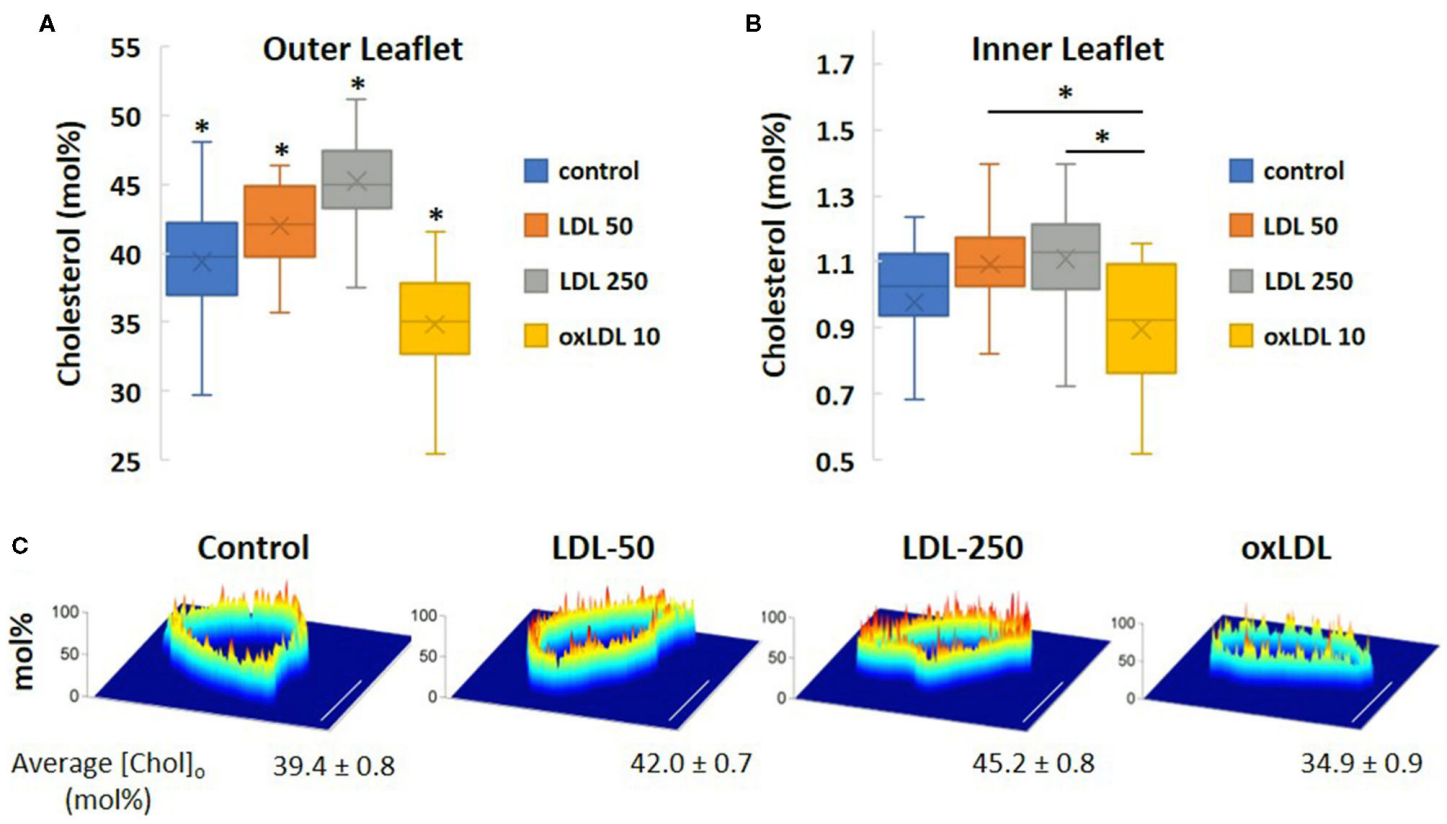
Simultaneous *in situ* quantification of changes in cholesterol content of LDL- and oxLDL-treated endothelial cells in the outer [Chol]_o_ **(A)** and inner [Chol]_i_ **(B)** membrane leaflets. **(C)** Spatially resolved quantifications of cholesterol in a cross-section of the outer cell membrane leaflet of representative cells by bio-orthogonal sensors. A pseudo-coloring scheme is used in each image (red: highest concentration; blue: lowest). Scale bars represent 10 μm. (*n* = 15-21 cells; *P < 0.05).

Using a novel bio-orthogonal cholesterol sensor for real-time measurement of cholesterol levels, we determined the impact of pro-atherogenic lipoprotein profiles on the asymmetric distribution of cholesterol in HAECs under the same conditions as described in Table 1. The biosensor sensor discriminated between the inner and outer membrane leaflets of HAECs *in situ* and determined cholesterol levels in each leaflet simultaneously. Using this sensor, we found that cholesterol is not only asymmetrically distributed between the leaflets, but is found predominantly in the outer leaflet, a result that is consistent with the observations by Liu et al. (10). Our current study is the first to employ this unique biosensor strategy to determine the impact of lipoproteins on cholesterol levels in endothelial membranes. Furthermore, we detected, for the first time, that exposure of cells to oxLDL resulted in a small but significant decrease in the cholesterol levels of both outer and inner membrane leaflets of HAEC plasma membranes (Figures 7A,B). LDL, however, had the opposite effect and increased the levels of cholesterol in both leaflets. The average cholesterol content values for the outer membrane leaflet (Figure 7A) and inner membrane leaflet (Figure 7B) for each treatment regime were obtained from spatially averaged values of the cholesterol mol% in the outer ([Chol]_o_) and inner ([Chol]_i_) membranes for each cell studied. In Figure 7C, images of spatially resolved quantifications of cholesterol in the outer cell membrane leaflet of representative cells for each treatment regime are shown. In each image, a pseudo-coloring scheme is used to spatially quantify cholesterol content, with red representing the highest concentration (in mol%) and blue the lowest. The average [Chol]_o_ content and standard deviation values for each treatment are given below each representative cell image.

## Discussion

This study addresses a fundamental question of the impact of LDL and oxLDL on the membrane structure and biophysical properties. A long-term controversy has existed in the field about whether exposure to LDL or oxLDL results in cellular cholesterol loading for both endothelial cells and for macrophages. Focusing on endothelial cells, our previous studies demonstrated that it is the exposure to LDL, and not oxLDL, that results in an increase in cellular cholesterol. Moreover, the actual increase in free endothelial cholesterol is rather subtle, even under high LDL concentrations that mimic severe hypercholesterolemia (7). In contrast, exposure to oxLDL does not lead to cholesterol increase, but instead results in an even more subtle increase in overall membrane concentrations of 7-ketocholesterol, a major oxysterol component of oxLDL. Although exposure to oxLDL increases the membrane concentration of 7KC by an order of magnitude (from ~0.2 to 2%) the levels of this sterol component remain subtle relative to the overall concentrations of other lipids in the membrane. These differences in the sterol compositions of LDL- vs. oxLDL-treated endothelial cells are associated with significant differences in lipid packing, as assessed by Laurdan imaging: while LDL increases lipid packing (7), as expected because of the increase in cholesterol, exposure to oxLDL results in the disruption of lipid packing (5). Furthermore, we have also demonstrated that the impact of oxLDL and 7-ketocholesterol on endothelial biomechanical properties are similar to those of cholesterol depletion, and can be reversed by cholesterol enrichment (5, 6, 8). In the current study, we investigated the mechanistic basis of this dichotomy in bilayers mimicking the physiological concentrations of free cholesterol and 7-ketocholesterol under normal and hypercholesterolemic conditions.

Our key findings are that, first, significant changes in lipid lateral diffusion and order are observed even with these very subtle changes in cholesterol and 7-ketocholesterol levels. Moreover, we found that, while both cholesterol and 7-ketocholesterol induce a decrease in the lipid lateral area and inhibit the lateral diffusion of all the lipid species tested in this study, cholesterol and 7-ketocholesterol have opposite effects on the lipid order parameters of all the lipids.

Previous studies have documented the membrane modulatory effects of exposure to high levels of 7-ketocholesterol. Concentrations of 7KC, up to 68% total sterol content, have been shown to alter membrane polarity and reduce lipid order (40, 41). Studies of liposomes composed of binary or tertiary mixtures of phospholipids and 7KC or cholesterol indicated that cholesterol increased lipid order and reduced polarity, while the effects of 7KC, including the extent of sterol tilt, were dependent on specific interactions with the surrounding phospholipids (42). We observed a small, but consistent, reduction in order across all lipid species in our 7KC simulation systems (Figure 6B), although our systems comparatively experienced a much smaller change in overall membrane composition, increasing from 0.24 to 2.1% 7KC.

Furthermore, Langmuir monolayer studies of binary mixtures of 7KC and DPPC showed that, compared with cholesterol/DPPC monolayers, 7KC had a weaker condensing effect and caused reduced interfacial rigidity—phenomena that were hypothesized to be explained by a possibly broader distribution of 7KC orientations within the monolayer as compared to cholesterol (43). In our simulations, we verified that there is indeed a broader distribution of 7KC tilt angles (Figure 5A) as compared to the cholesterol tilt distribution. We also showed that increasing 7KC content reduces the area/lipid (Figure 3—left) and lipid diffusivity (Figure 4C) to a small extent, both effects associated with membrane condensation and reduced interfacial rigidity, however, these effects caused by 7KC occur to a weaker extent than similar effects induced by cholesterol content increase. This finding is consistent with the observations of the Langmuir monolayer studies (43).

The overall effects of 7KC on membrane structure and dynamics are unexpected. In our study, we observed slight membrane condensation and reduced diffusivity, similar to cholesterol but weaker in extent, along with decreased lipid tail ordering, which is opposite to the increased ordering expected and observed for cholesterol. This perceived discrepancy can possibly be reconciled by the differences in orientation of these two sterol species within the membrane, thereby possibly affecting their interactions with surrounding lipid species. The larger 7KC tilt angles may cause surrounding lipid tails to become more disordered, while the smaller size of 7KC may induce effects on membrane condensation similar to cholesterol, but to a weaker extent. Indeed, in small simulated model bilayers containing 50:50 ratios of DPPC:6-ketocholesterol (6KC—a similar oxysterol to 7KC) vs. DPPC:cholesterol, differences in membrane modulation between the two systems were explained by the varying locations that the sterols occupied within the bilayer, with the 6KC ketone group moving up toward the polar water/lipid headgroup interface, while cholesterol remained in its expected membrane location (44). In that study, 7KC was predicted to exhibit similar behavior, which was later confirmed by Kulig et al. (38) in 128-molecule atomistic simulations of single-component POPC bilayers containing 10% sterol concentrations. All these previous studies, taken together, indicate that large concentrations of 7KC have a significant impact on membrane biophysical properties, however, the concentrations examined were much greater than the subtle physiologically relevant increases expected under hypercholesterolemic conditions. Our current study demonstrates that even subtle increases in 7KC or cholesterol levels in a multicomponent model bilayer can have distinct and significant effects on bilayer structure and dynamics and, consequently, membrane biophysical properties.

Using a novel cholesterol-sensitive sensor that discriminates between the inner and the outer leaflets of the membrane bilayer in live cells (10), we demonstrate that cholesterol distribution in the plasma membrane of HAECs is highly asymmetrical, with the outer leaflet having more than an order of magnitude higher cholesterol than the inner leaflet, as was previously shown by Liu et al. for different cell lines. This contrasts with the previous belief that cholesterol distribution between the leaflets is symmetrical, which was based on indirect evidence and energetic considerations. Indeed, Holthuis et al. showed that the distribution of lipids between membrane leaflets is a controlled process and is important for cell homeostasis (45). We also show that exposure to high levels of LDL results in small but significant increases in the level of cholesterol in both the outer and the inner leaflets, while maintaining the asymmetry, a feature that is proposed to play an important role in transmembrane signaling. This increase in membrane cholesterol is consistent with the results of our mass spectrometric analyses. We also observed a decrease in membrane cholesterol in cells exposed to oxLDL, which we did not observe using mass spectrometry. A major difference between the two methods is that the sensor is specific to the plasma membrane only, whereas mass spectrometry analyzes lipid extracts from all the cellular membranes.

We note that the measurements of cholesterol content using this novel bio-orthogonal sensor suggest that the total combined cholesterol content in both leaflets of the plasma membrane may be significantly lower than 40 mol%. As previously mentioned, the measurements from mass spectrometry are likely to be an overestimation since non-cholesterol and non-phospholipid membrane species are not considered. It is also possible that the biosensor underestimates the cholesterol content to some extent, for a variety of technical reasons, including partitioning of the sensor into the plasma membrane leaflets or cholesterol binding. We believe that the similarity of the trends in changes to cholesterol content between the two methods, showing significant, but very mild increases in cholesterol content in cells exposed to high levels of LDL, is most interesting. The obvious advantage of the mass spectrometry technique is that it provides information not only about cholesterol, but also oxysterols, for which there are currently no specific bio-orthogonal sensors. We chose to use cholesterol content values obtained from mass spectrometric analyses for our simulations because this method is frequently used to determine plasma membrane compositions in other studies (46–48). However, we also ran an additional set of simulations to determine if the same effects are observed at lower cholesterol concentrations, starting with a baseline of 30 mol% cholesterol and increasing it to 34 mol%, the same level of increase calculated from mass spectrometric analyses. We observed similar trends as those reported in this study in these additional simulations systems. For example, the measured area/lipid decreased from 47.9 ± 0.2 to 46.3 ± 0.2 Å^2^ for systems containing 30 and 34 mol% cholesterol, respectively. Further studies are needed to gain mechanistic insights into how pro-atherogenic lipoproteins alter the structure of the individual leaflets and dysregulate endothelial cell processes by altering the cholesterol distribution in endothelial membranes.

## Data Availability Statement

The raw data supporting the conclusions of this article will be made available by the authors, without undue reservation.

## Author Contributions

MA and IL conceived and designed research, interpreted results, and wrote the manuscript. MA performed experiments, ran simulations, analyzed data, and prepared figures. Both authors contributed to the article and approved the submitted version.

## Conflict of Interest

The authors declare that the research was conducted in the absence of any commercial or financial relationships that could be construed as a potential conflict of interest.
